# Expression of Semaphorins, Neuropilins, VEGF, and Tenascins in Rat and Human Primary Sensory Neurons after a Dorsal Root Injury

**DOI:** 10.3389/fneur.2017.00049

**Published:** 2017-02-21

**Authors:** Tomas Lindholm, Mårten Risling, Thomas Carlstedt, Henrik Hammarberg, Wilhelm Wallquist, Staffan Cullheim, Mattias K. Sköld

**Affiliations:** ^1^Department of Neuroscience, Karolinska Institutet, Stockholm, Sweden; ^2^Helsa Företagshälsovård Östermalm, Stockholm, Sweden; ^3^Hammersmith Hospital, University College London and Imperial College, London, UK; ^4^Department of Hand Surgery, Södersjukhuset, Stockholm, Sweden; ^5^Department of Clinical Science and Education, Karolinska Institutet, Södersjukhuset, Stockholm, Sweden; ^6^Department of Anesthesiology and Intensive Care, Västerås General Hospital, Västerås, Sweden; ^7^Department of Neuroscience, Section of Neurosurgery, Uppsala University, Uppsala, Sweden

**Keywords:** rhizothomy, DRG, regeneration, semaphorins, neuropilins, VEGF

## Abstract

Dorsal root injury is a situation not expected to be followed by a strong regenerative growth, or growth of the injured axon into the central nervous system of the spinal cord, if the central axon of the dorsal root is injured but of strong regeneration if subjected to injury to the peripherally projecting axons. The clinical consequence of axonal injury is loss of sensation and may also lead to neuropathic pain. In this study, we have used *in situ* hybridization to examine the distribution of mRNAs for the neural guidance molecules semaphorin 3A (SEMA3A), semaphorin 3F (SEMA3F), and semaphorin 4F (SEMA4F), their receptors neuropilin 1 (NP1) and neuropilin 2 (NP2) but also for the neuropilin ligand vascular endothelial growth factor (VEGF) and Tenascin J1, an extracellular matrix molecule involved in axonal guidance, in rat dorsal root ganglia (DRG) after a unilateral dorsal rhizotomy (DRT) or sciatic nerve transcetion (SNT). The studied survival times were 1–365 days. The different forms of mRNAs were unevenly distributed between the different size classes of sensory nerve cells. The results show that mRNA for SEMA3A was diminished after trauma to the sensory nerve roots in rats. The SEMA3A receptor NP1, and SEMA3F receptor NP2, was significantly upregulated in the DRG neurons after DRT and SNT. SEMA4F was upregulated after a SNT. The expression of mRNA for VEGF in DRG neurons after DRT showed a significant upregulation that was high even a year after the injuries. These data suggest a role for the semaphorins, neuropilins, VEGF, and J1 in the reactions after dorsal root lesions.

## Introduction

Primary sensory neurons represent a link between the peripheral nervous system (PNS) and the central nervous system (CNS). Among other things, they convey the crucial information needed for feedback and proper function of the motor systems. At spinal levels, the sensory axons enter the spinal cord *via* the dorsal roots, which mainly belong to the PNS. The primary sensory neurons are distributed to the dorsal root ganglions located in the distal part of the dorsal root. Hence, unlike other neurons in this pathway, they are located in the PNS and are often referred to as dorsal root ganglion neurons (DRG neurons). The DRG neuron have a rather unusual configuration with only one process—an axon that bifurcates and sends one peripheral branch into the peripheral nerve and one central branch to the CNS *via* the dorsal root. The response to injuries in these two axonal branches is highly dissimilar. Injury to peripheral branch initiates a powerful retrograde reaction in the cell body of the affected DRG neuron. This may initiate the death of the neuron, but surviving neurons have a capacity to regrow the peripheral branch. Injury to the central branch in the dorsal root seems to initiate a less vigorous reaction (1, 2). Thus, axon regrowth is possible in the PNS environment of the dorsal root, but the sprouts are typically arrested at the PNS–CNS border (3), and therefore, replantation of avulsed dorsal roots has not been considered to be useful even if recent studies have indicated that this situation can be changed by pharmacological intervention (4) or special procedures, such as removal of the DRG (5, 6). Due to this difference in response to injury, the DRG neurons offer the possibility to study the same neuron after two different kinds of injury where on is followed by regeneration (the peripheral injury) but the other one (central injury) followed by much less regenerative capacity.

In contrast, axons from spinal motoneurons have a high capacity for successful sprouting after lesions in the ventral funiculus of the spinal cord (7). These axons have been shown to penetrate CNS-type scar tissue inside the spinal cord, reenter the ventral root by crossing the CNS-PNS border, and regrow for long distances. This unusual regenerative capability has been employed for more practical use when avulsed ventral roots are replanted into the spinal cord, and this procedure has been shown to be followed by reinnervation of the ventral roots and functional recovery both in experimental animals (8–11) and clinical practice (12, 13).

In previous studies on ventral funiculus lesions or ventral root replantation, we have examined the expression of growth factors and a number of secreted and membrane-associated proteins demonstrated to affect axon steering, fasciculation, branching, or synapse formation through their action as chemorepellents and/or chemoattracants. These studies included members of the semaphorin family, the vascular and neuronal growth factor vascular endothelial growth factor (VEGF) and neuropilin 1 (NP1) and 2 and tenascins (14–16).

The semaphorins (SEMA) are secreted and transmembrane axon guidance molecules (17–19) that mediate axonal guidance in CNS and PNS in various ways including collapsing of growth cones (20) and also regulation of apoptosis (21) and neuroattractant capacities (22).

Semaphorin 3A (SEMA3A) (17), the prototype and founding member of the semaphorin family, has been characterized, besides ephrins, netrins, and slits, to function as a chemorepellent molecule with primarily inhibitory guidance capabilities (19, 23). During development, SEMA3A and its receptor proteins, NP1 (24–26), are known to take part in the regulation of axon fasciculation, axon guidance, and path finding. Another class 3 semaphorin, semaphorin 3F (SEMA3F) (27) has been shown to have widespread expression in adulthood and in sub regions of the CNS during embryogenesis. Neuropilin 2 (NP2) (24), which is the secreted receptor for SEMA3F, acts selectively to mediate repulsive guidance events in discrete populations of neurons and both ligand and receptor are expressed in strikingly complementary patterns during neurodevelopment (28). It has also been shown that a class 4 semaphorin, semaphorin 4F (SEMA4F), may play an important role in preventing growing retinal axons from deviating from their proper paths during development. In contrast to other SEMA, SEMA4F is expressed at the highest levels postnatal, and this might make it a potentially important molecule in nerve system maintenance and repair (29).

After intraspinal injuries to the ventral motoneuron axons, an injury known to be followed by successful regeneration of motoneuron axons (7), we did show increased expression SEMA3A in both injured motoneurons and spinal scar tissue (15), which indicates that SEMA3A expression could have in influence on the observed regenerative capacity of the motoneurons in this particular injury model.

Given this background, it appears logical to examine the expression of the same growth related genes in DRG neurons in a regenerative state (sciatic nerve lesion) or after a dorsal root lesion, which is not followed by functional regeneration.

## Materials and Methods

### Surgery and Collection of Tissues Concerning Animals

#### Dorsal Rhizotomy (DRT)

Adult female Sprague-Dawley rats (200–250 g) were anesthetized with intraperitonal administration of chloral hydrate (300 mg/kg) (KEBO-Lab, Sweden). A half-sided laminectomy was performed at the lumbar level, approximately at the L4 to S1 segments. The dural sac was cut open, and axotomy of two or three of the central processes of the dorsal roots was made with microsiccors (Fine Science Tools, Heidelberg, Germany). The wound was closed with sutures in multiple layers. The rats were allowed to survive for 1 day (*n* = 3), 3 days (*n* = 3), 5 days (*n* = 3), 7 days (*n* = 3), 14 days (*n* = 3), 21 days (*n* = 3), 42 days (*n* = 3), and 365 days (*n* = 3). Adult rats were deeply reanaesthetized and transcardially perfused with Tyrode’s solution. The pertinent tissues were rapidly dissected out and fresh frozen on dry ICE. Tissues from four adult rats were used as controls.

#### Sciatic Nerve Transection and Crush

Young, adult Sprague-Dawley rats (180–220 g; *n* = 3 per survival time) were anesthetized with chloral hydrate (300 mg/kg). After surgery, the animals were allowed to survive for 1, 3, 7, 14, 21, or 42 days. Tissue from four adult rats was used as controls.

##### Sciatic Nerve Transection and Resection

A 5–7 mm segment from the sciatic nerve was unilaterally resected below the obturator tendon. The wound was sutured to avoid contact between the proximal and distal ends.

##### Sciatic Nerve Crush (SNC)

The sciatic nerve was pressed one time with a pair of tweezers for 30 s, just below the obturator tendon. The wound was then inspected under a microscope to ensure that the crush was correctly performed.

The animals were killed with an overdose of pentobarbital (15 mg per 100 g body weight), and the L5–L6 dorsal root ganglions were taken out and frozen on a chuck.

### Embryonic Tissue

In addition to tissue from injured and non-injured adult rats, tissue from normal embryonic and new-born rats was used as positive controls due to the high levels of expression of the studied factors in embryonic and new-born tissue. Tissue from normal Sprague-Dawley rat embryos was obtained by killing pregnant female rats by CO2 overdose and collection by cesarean section at embryonic days 16 (E16, *n* = 1) or 18 (E18, *n* = 2). The first sperm-positive day of the dam was considered E0. In addition, new-born rats were anesthetized by hypothermia and killed by decapitation at postnatal day 0.5 (P0.5, *n* = 1) or postnatal day 4.5 (P4.5, *n* = 1). Noon of the day of delivery was considered P0.5. After decapitation the head, spinal cord and ventral root were rapidly fresh frozen as described above.

The use of animals for all experiments was approved by the local ethical committee for animal experimentation (Stockholms Norra Försöksdjursetiska Nämnd, N5/99, N366/01).

### Surgery and Collection of Tissues in Clinical Material

Cervical dorsal root ganglia whose roots were avulsed from the spinal cord were obtained in one female and four male patients (age range 18–44 years), all with traumatic injuries to their brachial plexus with delay between injury and collection of tissue at operation ranged between 1 day and 6 weeks. The ganglia were removed as a necessary part of the surgical repair procedure. In all cases, informed personal consent from each individual patient was obtained for tissue collection. Each ganglion was snap frozen in liquid nitrogen.

### *In Situ* Hybridization

Fresh-frozen DRG tissue was cut in an RNAse free environment on a cryostat (Microm HM 500 M, Heidelberg, Germany) in 14-μm-thick transverse sections from *Rattus norvegicus* thawed onto Probe-on object-slides (Fisher Scientific, Pittsburgh, PA, USA) and stored in black, sealed boxes at −70°C until used. Synthetic oligonucleotides were synthesized (CyberGene AB, Huddinge, Sweden). The sequence of the probes was checked in a GeneBank database search to exclude significant homology with other genes. The synthesized oligonucleotides were:

5′ TGG TCT CGC AGC ACT GAC ACC TCC CTC TCC AGC ATC TCG ATT CGG CTC AA 39, complementary to nucleotides 3,274–3,323 of the mRNA encoding *Rattus norvegicus* J1-160/180 mRNA (GenBank Accession No. Z18630);
ACA AAG GCC GGG GCA CTC TCA AGG GAG CAG CAA CAA GTG GAA GCA CAT GC, which is complementary to nucleotides 2,205–2,254 of the Rattus norvegicus mRNA for semaphorin III/collapsin-1 (Genebank accession X95286);
GGG GTC TGG GCT CAG GGG AGG GGA AGT CAC AAA TGC AGC TGC CTT GGC CC, complementary to nucleotides 889–938 of the mRNA for the Rattus norvegicus collapsin response mediator protein (Genebank U52095);
5′ AGC AGA CGA GCC GCG CCT TCA GGA ATG TGC TCC ACT TGT TGA CCA GGC AA 3′ complementary to nucleotides 1,143–1,192 of Homo sapiens SEMA3F, mRNA (Genbank accession HSU38276), which is 97% identical with Mus musculus, semaphorin 3 F;
5′ CAG ATC CTC CAA GAC ACT GAG CTG AGC TCC AAT GCG CAC AGC CCG GTG GA 3′ complementary to nucleotides 1,475–1,524 of Rattus norvegicus (SEMA4F), mRNA (Genbank accession NM_019272.1);
5′ TGG GCC AGG ATG CAC TCT GAG CAG CTC TGG AGA CGG CCA CAG TTG GTT GT 3′ complementary to nucleotides 1,079–1,128 of Homo sapiens semaphorin 4F mRNA (Genbank accession NM_004263.1);
5′ AAC AGG CAC AGT ACA GCA CGA CCC CAC AGA CAG CCC CCA GGA GGA CCC CC 3′ complementary to nucleotides 2,601–2,650 of Homo sapiens NP1 mRNA (Genbank accession XM_005798.2);
GCA CAA CTC CAC AGA CTG CAC CCA GGA GCA CCC CCA GGG CAC TCA TGG CT complementary to nucleotides 2,580–2,629 of Rattus norvegicus neuropilin mRNA (Genebank AF018957);
CCA CGT CTG CGG GCG GAT CCT GAT GAA ACG AGT CAA CAG CGG CGT GTG CA complementary to nucleotides 1,504–1,553 of Rattus norvegicus neuropilin-2 mRNA (Genebank AF016297);
5′ GTC TGT CCA GTC ACA GCC CAG CAC CTC CAG CCG CAT CCC AAT CCC CGC CG 3′ complementary to nucleotides 1,739–1,788 of Homo sapiens NP2 mRNA (Genbank accession XM_002670.2);
5′ CTG GGG CTG GGG GCG GTG TCT GTC TGT CTG TCC GTC AGC GCG ACT GGT CA 3′ complementary to nucleotides 157–206 of Homo sapiens VEGF mRNA (Genbank accession AF022375.1);
5′ TCG ACG GTG ACG ATG GTG GTG TGG TGG TGA CAT GGT TAA TCG GTC TTT CC 3′ complementary to nucleotides 365–414 of the mRNA encoding rat VEGF (GeneBank accession AF062644).

The probes were labeled at the 3′-end with deoxyadenosine- alpha-(thio)triphosphate -35S- (NEN, Boston, MA, USA) by using terminal deoxynucleotidyl transferase (Amersham Pharmacia Biotec, Uppsala, Sweden) and hybridized to the sections, without pretreatment, for 16–18 h at 42°C. The hybridization mixture contained: 50% formamide (G.T. Baker Chemicals B W, Deventer, The Netherlands), 4 × SSC (1 × SSC is 0.15 M NaCl and 0.015 M sodium citrate), 1 × Denhardt’s solution (0.02% each of polyvinyl-pyrrolidone, bovine serum albumin and Ficoll), 1% Sarcosyl (*N*-lauroylsarcosine; Sigma-Aldrich), 0.02 M phosphate buffer (pH 7.0), 10% dextran sulfate (Amersham Pharmacia Biotec), 500 µg/ml sheared and heat-denatured salmon sperm DNA (Sigma-Aldrich), and 200 mM dithiothreitol (DTT; Sigma-Aldrich). Following hybridization, the sections were washed several times in 1 × SSC for 15 min at 60°C, rinsed in distilled water, and dehydrated in ascending concentrations of ethanol. The sections were then coated with NTB2 nuclear track emulsion (Kodak, Rochester, NY, USA). After 3–5 weeks, the sections were developed in D-19 developer (Kodak) for 5 min at room temperature and fixed in AL-4 fixative (Kodak) for 5 min. Finally, the slides were counterstained with cresyl violet (Sigma C5042, USA) and then dehydrated in ascending concentrations of ethanol, mounted in Entellan (Histolab products AB, Göteborg, Sweden), and coverslipped.

### Image Analysis

The hybridization signal was recorded with a 40× objective in a Leica DM RBE microscope equipped with a dark-field condenser (Leica, Wetzlar, Germany) and digitized at a final linear magnification of 400× using a Kappa video camera (Mikroskop System, Näsviken, Sweden) and a Perceptics PixelBuffer image grabber card (Parameter AB, Stockholm, Sweden) mounted in an Apple Macintosh computer (Apple Inc., USA). The gray scale of the darkfield image was adjusted and segmented by using the “enhance contrast” and “density slicing” features of the NIH Image software (version 1.55), National Institutes of Health Image software (version 1.55, Bethesda, MD, USA). After that the contour of the cell-soma had been outlined manually, the density of silver grains over neuronal profiles in the dorsal root ganglia could be assessed automatically. Cells having a hybridization signal of three times the background level or higher were considered positive. For each neuron studied, separate recordings of the area of the soma and the area covered by silver grains were obtained. These data allowed for a calculation of labeling intensity (particle density), over each analyzed neuron. Six spinal cord sections, derived from all three of the animals in each experimental group, were analyzed. They were randomly selected, but in a few cases, sections were excluded due to artifacts. Statistical evaluation of the counts was performed using Prism 2.0 (GraphPad Inc., USA) software. Images were sampled directly from the microscope, using a Nikon 950 and 990 digital camera (Bergström Instrument AB, Solna, Sweden). Representative digital images were mounted with Adobe InDesign software (Adobe Systems Inc., USA) and used for illustration.

### Statistics

When comparing the density, in series with three or more different animals or humans, of the silver grains located to neurons in the affected sides DRG’s, we have used the one-way ANOVA Kruskal–Wallis statistics (Dunn’s Multiple Comparison Test). When it has been only two humans we have used the Mann–Whitney’s *t*-test.

## Results

The embryonic tissue was used as a positive control of the different mRNA probes and expression patterns similar to what has previously described was found (25, 29–32).

Examination of sections incubated with the radiolabeled SEMA3A antisense probe showed that many, but not all, DRG neurons in both rats and humans had a strong labeling signal (Figure 2). Image analysis revealed that there was a trend for down regulation of SEMA3A mRNA in the DRG of rats subjected to dorsal rhizotomy. The expression of SEMA3A mRNA reached it lowest level at 21 days after the injury. The mean particle density (i.e., the fraction of the area of the examined DRG neurons that was covered by silver grains) was about 4.6% at this stage, to be compared with 18.5% in control DRG neurons. These values were obtained by recording labeling density in about 100 neurons that were randomly selected in three different rats at each survival time. Although, it may be argued that the measurements are not independent, these recorded values from individual neurons were analyzed using one-way ANOVA Kruskal–Wallis statistics (Dunn’s Multiple Comparison Test), which indicated that SEMA3A mRNA was significantly down regulated in the DRG (*P* < 0.001) 3, 7, and 21 days after the dorsal root injury. The labeling was gradually restored and reached a mean value of 18.7% 1 year after the operation. Thus, at 1 year after the trauma, there was no significant difference between control and experimental DRG (Figure 2A). This transient down regulation in the labeling intensity was most pronounced in the small DRG neurons (Figure 2D). Examination of sections from rats subjected to sciatic nerve transection (SNT) or SNC showed that the labeling signal for SEMA3A in the DRG was largely unaltered after these injuries. Dunn’s test indicated a transient upregulation of the SEMA3A signal 3 days after SNT but not after SNC. The signal was normalized 14 days after the injury (Figure 2B). The labeling intensity in DRG from patients who had sustained root avulsion injury was similar to what had been observed in rats (Figures 2A,C).

In sections from normal rat DRG that had been hybridized with a SEMA3F antisense probe, there was a significant labeling signal in virtually all DRG neurons. We found a trend for down regulation of labeling with the radiolabeled SEMA3F antisense probe in rats subjected to dorsal root transection (Figure 3A). A decrease in mean labeling was observed from day 5 and reached the lowest value at 3 weeks (*P* < 0.001) after the operation. The labeling signal was then gradually restored and was completely restored 1 year after the operation (Figure 3A). The labeling for SEMA3F was significantly upregulated in all rats subjected to sciatic nerve lesions (Figure 3B). Similar trends were observed in sections hybridized with a SEMA4F antisense probe. Thus, a transient downregultion was observed in rats subjected to dorsal root lesion (Figure 3C), whereas a transient upregulation in the labeling signal for SEMA4F could be detected in rats subjected to SNT or SNC (Figure 3D). Labeling with the probe for human SEMA4F in sections of DRG from patients after root avulsion seemed to correspond fairly with the findings in rats (Figures 3C,E) with regard to intensity and distribution.

**Figure 1:**
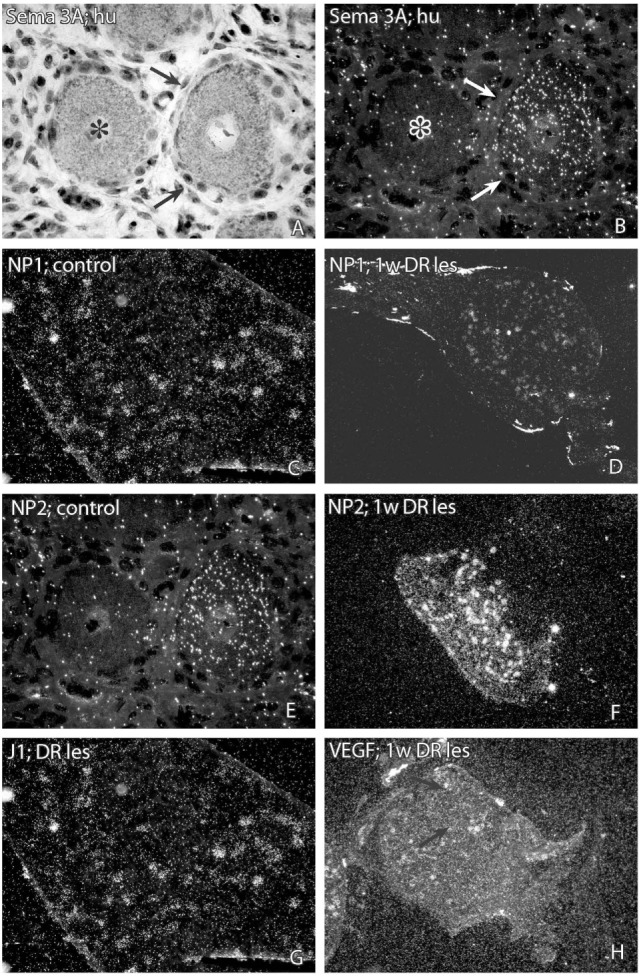
**Photomicrographs showing representative sections from dorsal root ganglia after *in situ* hybridization for detection of semaphorin 3A (SEMA3A) (A,B), neuropilin 1 (NP1) (C,D), neuropilin 2 (NP2) (E,F), J1 (G), or vascular endothelial growth factor (VEGF) mRNA (H)**. **(A)** (=bright field) and **(B)** (=dark field), two neurons in a DRG from a patient treated for root avulsion injury is shown. In dark field illumination it is possible to see that the neuron indicated with arrows has a positive labeling signal for SEMA3A, whereas the neuron indicated by an asterisk is unlabeled. Panel **(C)** is a low magnification micrograph showing a rat DRG hybridized with a NP1 antisense probe. Panel **(D)** shows a section from a DRG one week after dorsal root transection. The labeling signal for NP1 is clearly upregulated in Panel **(D)**. Panels **(E,F)** show DRG from a control rat **(E)** and a rat subjected to dorsal root transection **(F)** after hybridization with a NP2 antisense probe. The labeling signal for NP2 was clearly higher after dorsal root transection **(F)** than in the control DRG **(E)**. The micrograph 1G shows a section from a rat DRG 1 week after dorsal root transection after hybridization with a J1 antisense probe. A number of neurons displayed a positive labeling for J1, although at a similar level as in control rats. The micrograph 1H shows a section from a rat DRG 1 week after dorsal root transection. The small neuron that is indicated by the arrows had a fairly high labeling signal for VEGF mRNA.

In sections from DRG of normal rats incubated with the NP1 antisense probe, there was a detectable labeling signal in many of the DRG neurons (Figure 1). This signal was found to be clearly up regulated both after dorsal root lesion (Figures 1 and 4A) and after sciatic nerve injury (Figure 4B). This upregulation did not seem to be specific for any size-class of DRG neurons (Figure 4C). With exception for rats surviving for 42 days after SNC, the difference between normal and operated rats was significant at all survival times according to Dunn’s test. Almost identical results were obtained with the probe for NP2. Thus, a large number of the DRG neurons in control rat ganglia had a significant labeling for NP2 (Figure 1), and this signal was clearly upregulated in rats subjected to dorsal root transection (Figure 1) or sciatic nerve injury. With exception for rats surviving for 42 days after SNC, the labeling signal was elevated at all survival times both after dorsal root lesion and sciatic nerve lesions (Figures 5A,B) and the changes did not appear to be size specific (Figure 5C).

**Figure 2:**
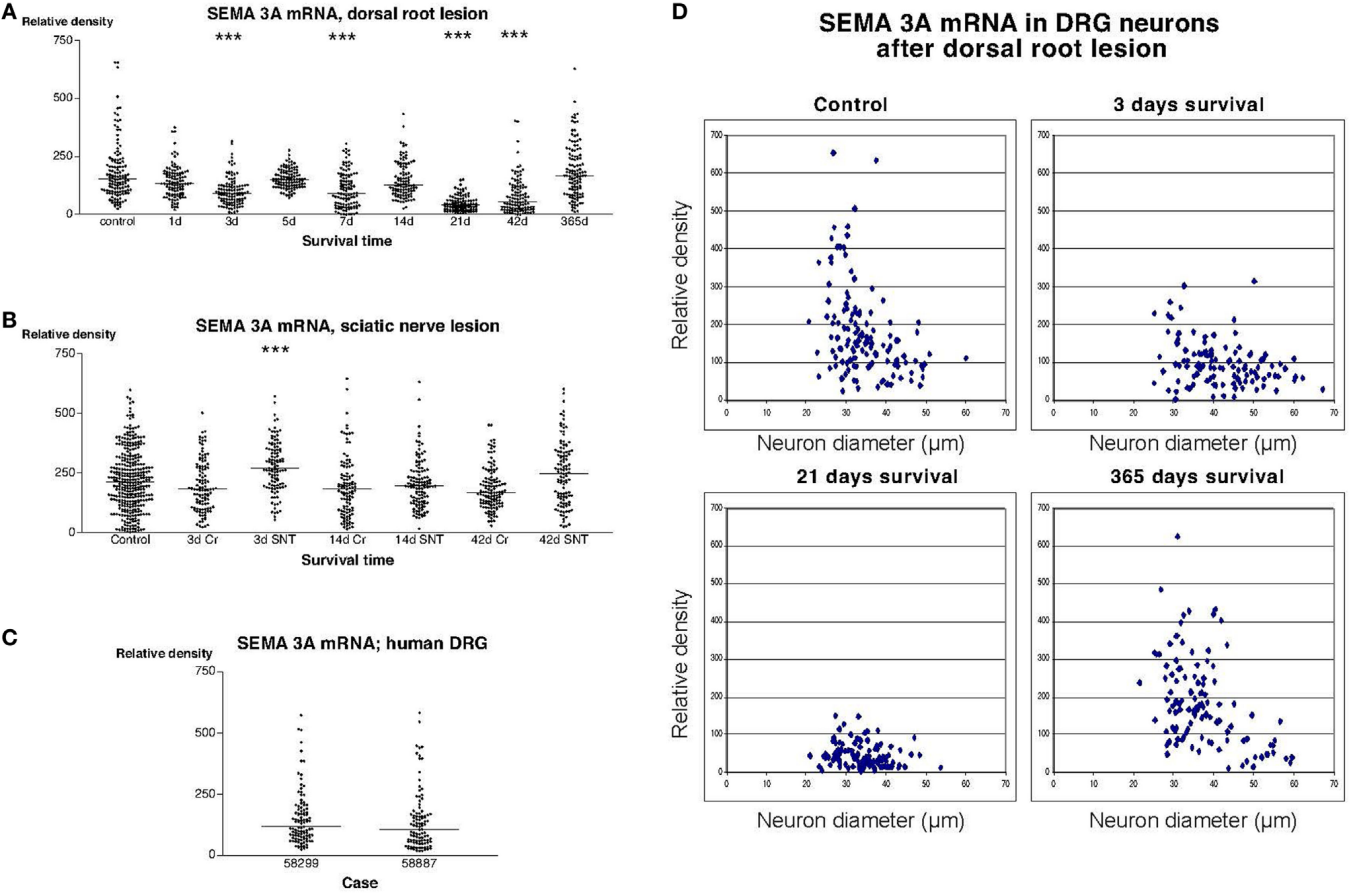
**(A–C)** illustrate the relative density of labeling for semaphorin 3A (SEMA3A) mRNA in dorsal root ganglion neurons at different survival times (expressed in days = d) after dorsal root transection **(A)** or sciatic nerve transection (=SNT) or sciatic nerve crush (=Cr) **(B)**. Each dot represents an analyzed neuron and a horizontal bar indicates the median density at each survival time. The asterisks refer to results obtained with one-way ANOVA Kruskal–Wallis statistics (Dunn’s Multiple Comparison Test; ***a difference between controls and the experimental group that is significant according to the test; *P* < 0.001). Panel **(A)** illustrates that there was a transient down regulation in the expression of SEMA3A mRNA after dorsal root transection, whereas the expression of SEMA3A was largely unchanged after sciatic nerve injury **(B)**. Panel **(C)** is a density plot for SEMA3A mRNA in human dorsal root ganglion neurons in two patients who had sustained root avulsion injury. Horizontal bar indicates the median density. **(D)** The diameter (expressed in microns) of the examined dorsal root ganglion neurons (DRG) has been plotted against the labeling density for SEMA3A mRNA in control rat DRG and at different survival times after dorsal root transection. Each examined neuron is represented by a dot in the diagram. In the control diagram is shown that many of the small DRG neurons had a high labeling density. The observed down regulation in SEMA3A after dorsal root transection appeared to affect the small neurons more profoundly than the larger ones. One year after the injury, the size distribution of the labeled neurons was found to be largely restored.

**Figure 3:**
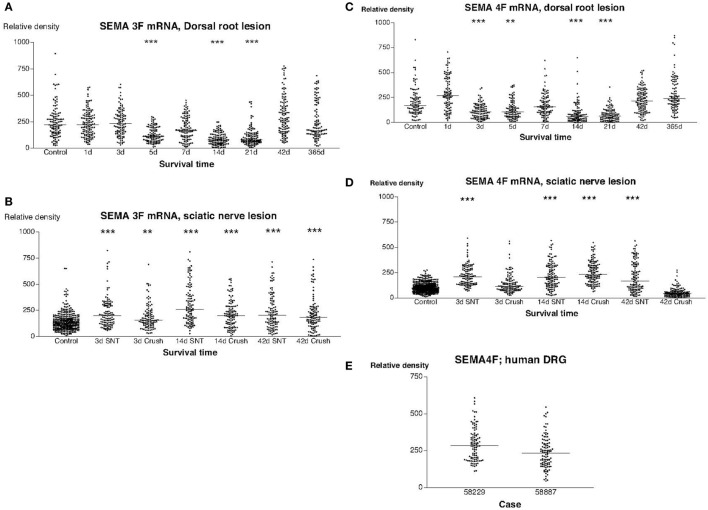
**This diagram illustrates the relative density of labeling for semaphorin 3F (SEMA3F) and semaphorin 4F (SEMA4F) mRNA in dorsal root ganglions neuron at different survival times (expressed in days = d) after dorsal root transection (A) or sciatic nerve transection (=SNT) or sciatic nerve crush (SNC) (=Cr) (B)**. Each dot represents an analyzed neuron and a horizontal bar indicates the median density at each survival time. The asterisks refer to results obtained with one-way ANOVA Kruskal–Wallis statistics (Dunn’s Multiple Comparison Test; ***a difference between controls and the experimental group that is significant according to the test; *P* < 0.001). Panel **(A)** illustrates that there was a transient down regulation in the expression of SEMA3F mRNA after dorsal root transection whereas the expression of SEMA3F was significantly upregulated after sciatic nerve injury **(B)**. Panels **(C,D)** illustrate the relative density of labeling for SEMA4F mRNA in dorsal root ganglion neurons at different survival times (expressed in days = d) after dorsal root transection **(C)** or sciatic nerve transection (=SNT) or SNC (=Cr) **(D)**. Each dot represents an analyzed neuron and a horizontal bar indicates the median density at each survival time. The asterisks refer to results obtained with one-way ANOVA Kruskal–Wallis statistics (Dunn’s Multiple Comparison Test; ***a difference between controls and the experimental group that is significant according to the test; *P* < 0.001). Panel **(C)** illustrates that there was a transient down regulation in the expression of SEMA4F mRNA after dorsal root transection whereas the expression of SEMA4F showed a transient upregulation after sciatic nerve injury **(D)**. Panel **(E)** is a density plot for SEMA4F mRNA in human dorsal root ganglion neurons in two patients who had sustained root avulsion injury. Horizontal bar indicates the median density.

**Figure 4:**
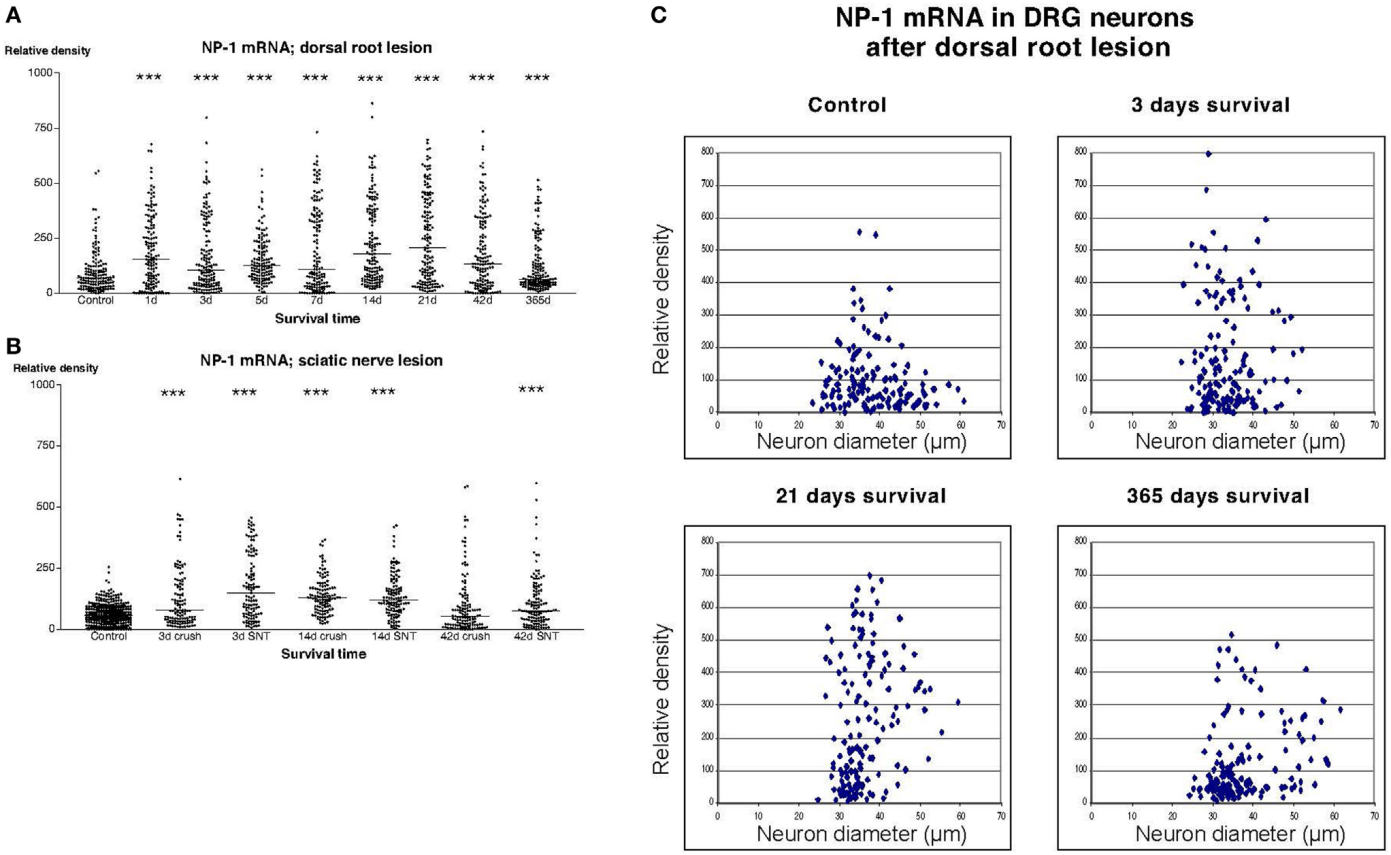
**Diagram illustrating the relative density of labeling for neuropilin 1 (NP1) mRNA in dorsal root ganglion neurons at different survival times (expressed in days = d) after dorsal root transection (A) or sciatic nerve transection (=SNT) or sciatic nerve crush (=Cr) (B)**. Each dot represents an analyzed neuron and a horizontal bar indicates the median density at each survival time. The asterisks refer to results obtained with one-way ANOVA Kruskal–Wallis statistics (Dunn’s Multiple Comparison Test; ***a difference between controls and the experimental group that is significant according to the test; *P* < 0.001). There was a transient upregulation in the expression of NP1 mRNA after dorsal root transection [panel **(A)** and after sciatic nerve injury panel **(B)**]. **(C)** The diameter (expressed in microns) of the examined dorsal root ganglion neurons (DRG) has been plotted against the labeling density for NP1 mRNA in control rat DRG and at different survival times after dorsal root transection. Each examined neuron represents a dot in the diagram. The observed upregulation in NP1 after dorsal root transection appeared to affect neurons in all size classes.

**Figure 5:**
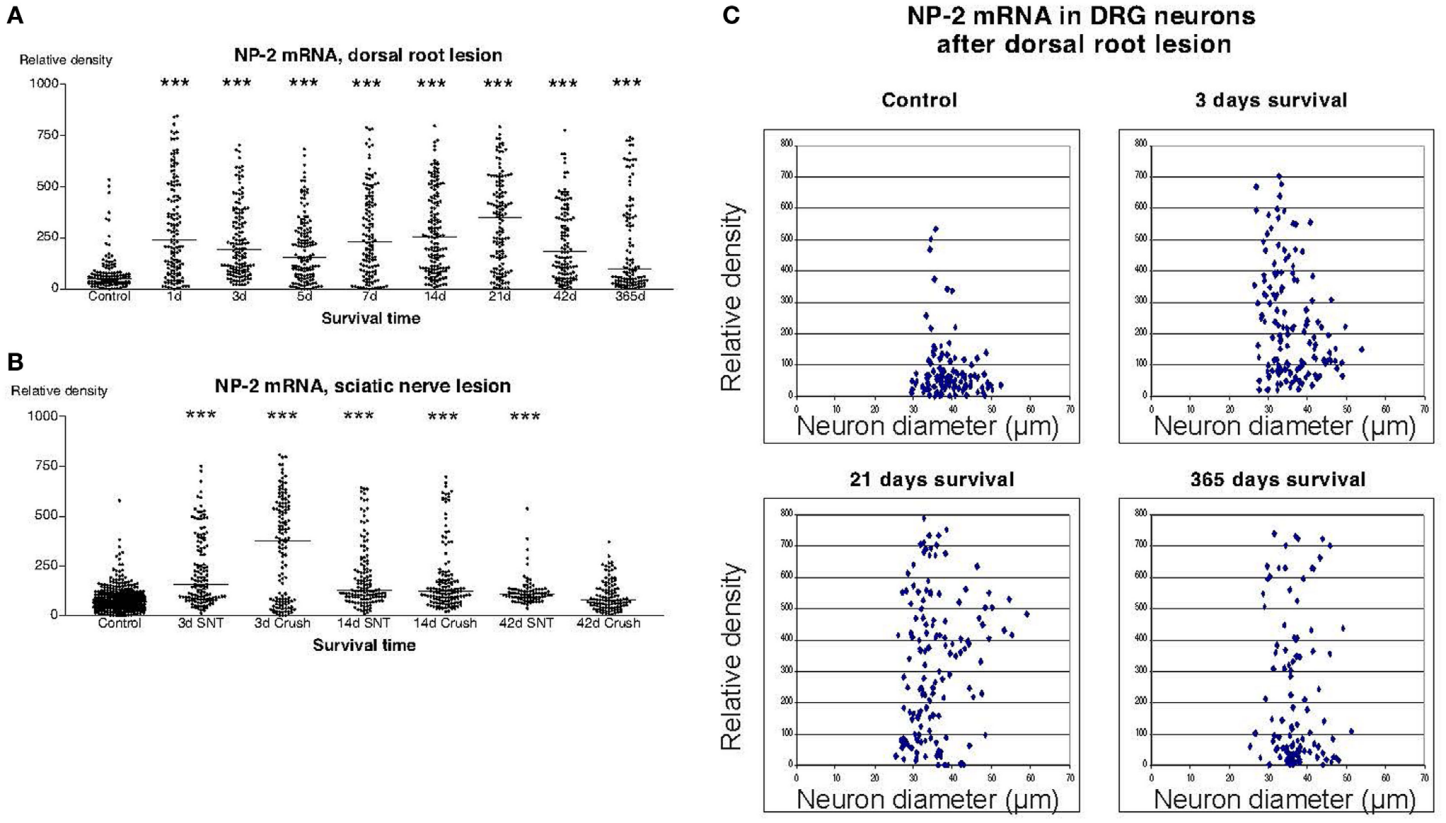
**Diagram illustrating the relative density of labeling for neuropilin 2 (NP2) mRNA in dorsal root ganglion neurons at different survival times (expressed in days = d) after dorsal root transection (A) or sciatic nerve transection (=SNT) or sciatic nerve crush (SNC) (=Cr) (B)**. Each dot represents an analyzed neuron and a horizontal bar indicates the median density at each survival time. The asterisks refer to results obtained with one-way ANOVA Kruskal–Wallis statistics (Dunn’s Multiple Comparison Test; ***a difference between controls and the experimental group that is significant according to the test; *P* < 0.001). Panel **(A)** illustrates that there was a transient upregulation in the expression of NP2 mRNA after dorsal root transection. The expression of NP2 in DRG neurons was also found to be increased after sciatic nerve injury **(B)**. **(C)** The diameter (expressed in microns) of the examined dorsal root ganglion neurons (DRG) has been plotted against the labeling density for NP2 mRNA in control rat DRG and at different survival times after dorsal root transection. Each examined neuron represents a dot in the diagram. The observed upregulation in NP2 after dorsal root transection could be observed in neurons of all size classes.

**Figure 6:**
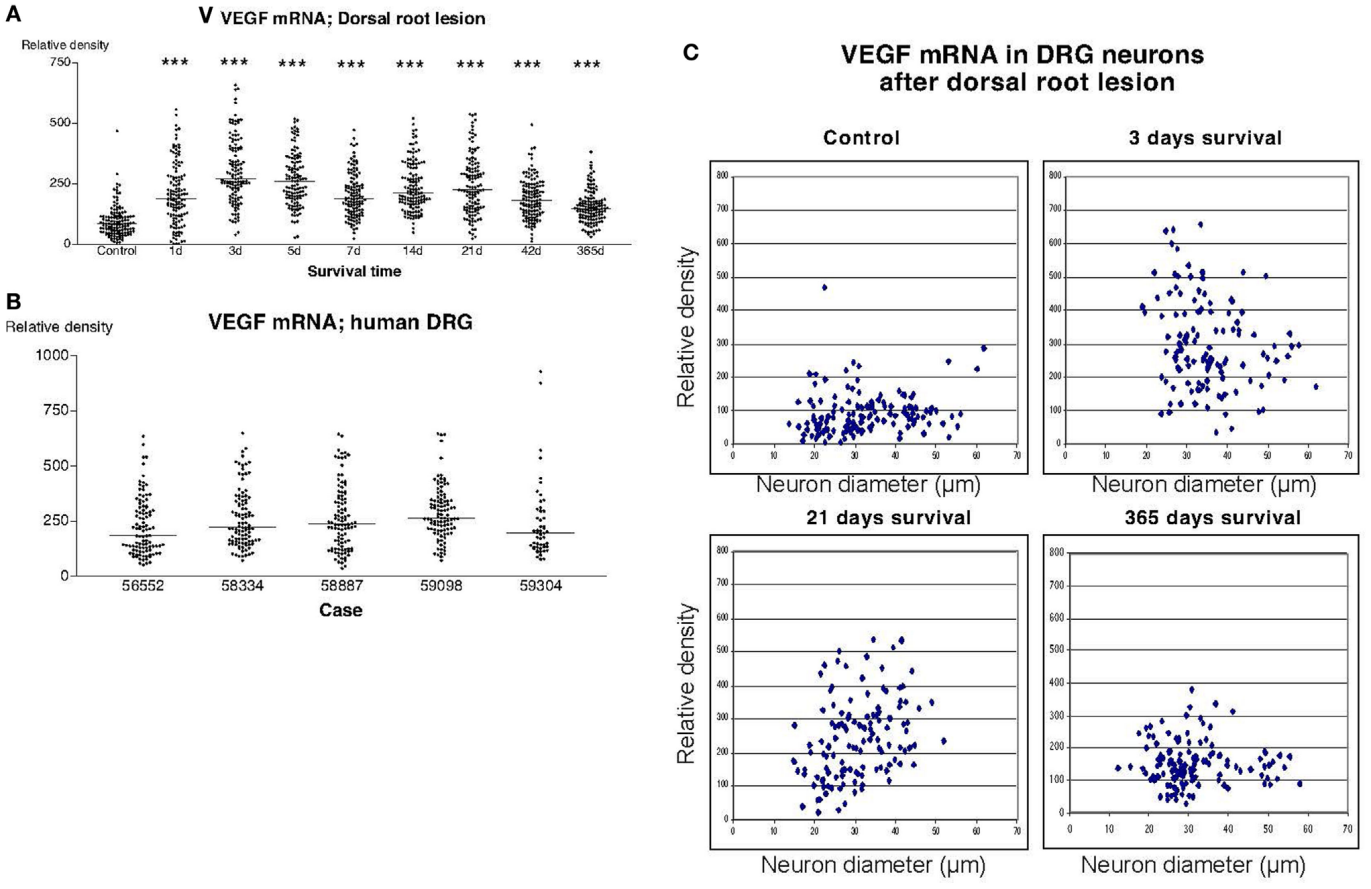
**The diagram shown in panel (A) illustrates the relative density of labeling for vascular endothelial growth factor (VEGF) mRNA in dorsal root ganglion neurons at different survival times (expressed in days = d) after dorsal root transection**. Each dot represents an analyzed neuron and a horizontal bar indicates the median density at each survival time. The asterisks refer to results obtained with one-way ANOVA Kruskal–Wallis statistics (Dunn’s Multiple Comparison Test; ***a difference between controls and the experimental group that is significant according to the test; *P* < 0.001). There was a distinct increase in the expression of VEGF mRNA after dorsal root transection. Panel **(B)** is a density plot for VEGF mRNA in human dorsal root ganglion neurons from five different patients who had sustained root avulsion injury. It can be revealed that the labeling in these patients had a similar intensity as the labeling that was observed in rats subjected to dorsal root transection. **(C)** The diameter (expressed in microns) of the examined dorsal root ganglion neurons (DRG) has been plotted against the labeling density for VEGF mRNA in control rat DRG and at different survival times after dorsal root transection. Each examined neuron is represented by a dot in the diagram. The observed upregulation in VEGF after dorsal root transection appeared to affect neurons of all sizes.

Also in sections hybridized with the VEGF probe, there was a significant upregulation of the labeling signal at all survival times after dorsal root lesion (Figure 6A). Although, still significantly upregulated according to Dunn’s test, there seemed to be gradual normalization in the labeling 1 year after the operation. The upregulation of labeling for VEGF affected neurons of all sizes (Figure 6C). The labeling for VEGF in DRG of patients treated for avulsion injury (Figure 6B) was similar to the findings in rats subjected to dorsal root lesion.

**Figure 7:**
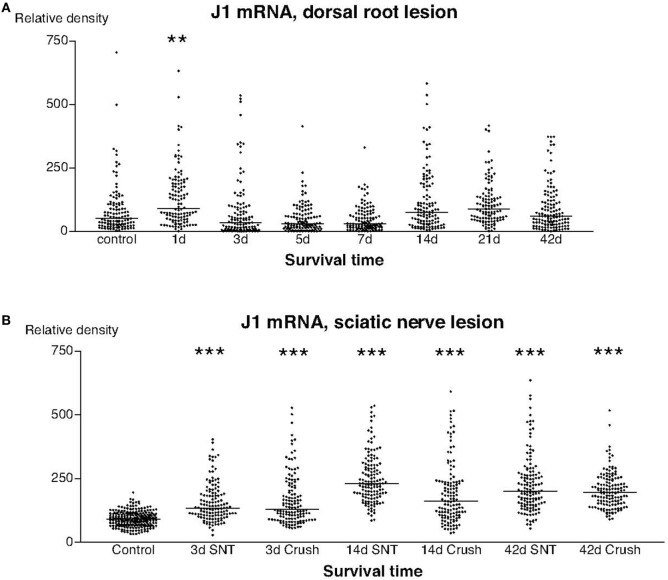
**This diagram illustrates the relative density of labeling for J1 mRNA in dorsal root ganglion neurons at different survival times (expressed in days = d) after dorsal root transection (A) or sciatic nerve transection (=SNT) or SNC (=Cr) (B)**. Each dot represents an analyzed neuron and a horizontal bar indicates the median density at each survival time. The asterisks refer to results obtained with one-way ANOVA Kruskal–Wallis statistics (Dunn’s Multiple Comparison Test; ***a difference between controls and the experimental group that is significant according to the test; *P* < 0.001). Panel **(A)** illustrates that the expression of J1 mRNA was largely unchanged after dorsal root transection, whereas the expression of J1 was clearly upregulated after sciatic nerve injury **(B)**.

The labeling for J1 mRNA showed two different patterns. With the possible exception for the first postoperative day, there were no detectable changes in the labeling for J1 after dorsal root transection (Figure 7A), whereas there was a significant upregulation in the signal for J1 at all examined stages after sciatic nerve injury (Figure 7B).

## Discussion

In this study, we do investigate the expression of SEMA, neuropilins, and tenascin in different injury models to the dorsal spinal roots. The injuries are either applied to the central axon of the dorsal root (DRT), i.e., the root central to the dorsal root ganglion (DRG), or to the peripheral axon [sciatic nerve transection (SNT) and SNC], the part peripheral to the DRG. These two different injuries to the same neuron results in different regenerative responses, making them interesting models for the study of nerve regeneration and thus for study of nerve guidance molecules, such as SEMA. After an injury to the central axon of DRG neurons, the axons are less able to regenerate then after injuries to the peripheral axons of the DRG neurons that instead are followed by strong regenerative capacity (1, 2). This interesting difference has been studied in various ways, and it has for example been shown that the growth associated protein GAP-43, a molecular marker for regenerative response after nerve injury (33) is upregulated in DRG neurons after an injury to the peripheral DRG axon but not to the central DRG axon (34). Interestingly, the regeneration of the central DRG axon can be enhanced by concurrent injury to the peripheral DRG axon (2, 35–37), and such injuries do result in cellular responses in DRG neurons typical for a regenerative state, including induction of GAP-43 (34). These kind conditional injuries can also support regeneration of dorsal root axons to enter the spinal cord (3).

Another marker for regenerative responses after nerve injury, activating transcription factor 3 has been studied after injuries to DRG axons and do show a pattern similar to GAP-43 with a strong upregulation in DRG neurons after peripheral axon injury but a much less pronounced expression after central DRG axon injury (38).

We have also previously studied the expression of SEMA and VEGF in an injury model where motoneuron axons are cut in the ventral funiculus within the spinal cord (15, 16, 39). This is an injury model followed by successful regeneration of the injured motoneuron axons through the scar tissue and in to ventral roots (7), which enables us to compare the expression pattern of the SEMA in the present study with the expression of the same factors in a model with successful regeneration.

We show in this study that mRNA for SEMA3A in the DRG neurons was significantly downregulated after a DRT and that its receptor, NP1, showed an instant mRNA upregulation in the DRG following DRT, SNT, and SNC, the latter being opposite to findings from Gavazzi and colleagues who reported an upregulation of NP1 in DRG after SNT but no changes in NP1 mRNA after DRT (40). If considering that DRT is followed by a less vigorous regrowth of axons, it is reasonable to speculate that the down regulation of SEMA3A as shown by us reflects that SEMA3A could be of importance for nerve regrowth in injured DRG. Decreased expression of SEMA3A in motor and sensory neurons during peripheral nerve regeneration has indeed been discussed as a molecular event that is part of the adaptive response related to the success of regenerative neurite outgrowth occurring peripheral nerve injury (41). We have in a previous publication also described increased levels of SEMA3A in both neurons and scar tissue in a model followed by successful nerve regeneration of motoneuron axons (15) making the described down regulation of SEMA3A in a model followed by less successful nerve regeneration interesting. Others have also demonstrated that upregulation of SEMA3A, SEMA3F, NP1, and NP2 are correlated with regrowth in peripheral nerve injuries where expression of these factors were found mainly in Schwann cells distal of the injury (42, 43), again pointing toward possibly positive nerve growth guidance capacities of SEMA3A.

On the other hand, do we in this study not find a consistent upregulation of SEMA3A after SNT and SNC, SNT 3d postoperatively being an exception, see Figure 2B, even though these kind of injuries are known to be followed by nerve regeneration (1). SEMA3A is secreted and not membrane bound, which could be of importance for the interpretation of our findings. It might not be that the expression of SEMA3A we find have an impact on the DRG neurons directly but rather on peripheral targets such as the dorsal horn where others have found increased expression of the SEMA3A receptor NP1 after dorsal root rhizotomy (44). In this way, the secreted SEMA3A could possibly interact with NP1 at the dorsal horn and be a part in the well described inhibition of regenerating DRG neuritis over the CNS-PNS border of the dorsal horn (3).

Our findings show a striking trend for downregulation of mRNA for SEMA3F during the examined period after DRT, with a decrease from 42 days and normalization at 1-year post-trauma. On the other hand, did SEMA3F mRNA show an early significant upregulation after SNT. The former finding do correspond to our findings on dorsal root injury and downregulation of SEMA3A as shown in Figure 2, while the latter do not correspond to the findings of unchanged SEMA3A levels after sciatic injury. We have previously described that mRNA for SEMA3F has a strong expression in the ventral root on the injured side after a ventral funiculus lesion in adult rats (15), thus in a model followed by successful regeneration, which might indicate that the downregulation shown after DRT reflects the weak regeneration shown after this injury. We observed a significant downregulation of mRNA for SEMA4F in the DRG neurons after a DRT. On the other hand, in the same time, the labeling of mRNA for SEMA4F was instantly higher in the DRG following SNT and SNC. This implicates a role in the post-traumatic regenerative response of adult axotomized DRG neurons.

Vascular endothelial growth factor is a secreted mitogen with importance in regulation of angiogenesis and vascular permeability. Induction of VEGF has been reported both after traumatic spinal cord injuries (16). It has been shown that VEGF do have a direct neurotropic/neuroprotective function (45, 46). For example, Sondell and coworkers have shown that VEGF_165_ could stimulate axon outgrowth from DRG *in vitro* (45). It is known that the neuropilin receptors 1 and 2, NP1 and NP2, are not only receptors for the SEMA but does also function as co-receptors for VEGF_165_ (47) and are as such of importance for the VEGF mediated rearrangement of the actin skeleton in the nerve growth cone (48, 49). Thus, the neuropilins are receptors for two unrelated ligands: SEMA acting as inhibitors of axon growth and VEGF acting as an angiogenic and neurotropic factor. The interplay between VEGF and SEMA are not yet fully understood, but it has been shown that VEGF_165_ and SEMA do compete for the binding sites of NP1 (50). In this work, we did also find a strong upregulation of VEGF mRNA in DRG neurons after dorsal root lesions. We did also find an upregulation of the VEGF co-receptors NP1 and NP2 mRNA that coincide in time with the upregulation of VEGF. In addition, SEM3A mRNA is promptly downregulated during the same time. Since VEGF and SEMA3A both binds to the NP receptors (47), this could imply that there is an interaction between VEGF and SEMA3A *in vivo* in our injury model system and that VEGF could compete with SEMA3A in the binding to the NP receptors. This, in turn, could have a positive impact on the axon growth from DRG neurons after dorsal root lesions. Others have shown both that VEGF and SEMA do compete for the binding site of NP1 (50) and that VEGF_165_ do inhibit the action of SEMA3A *in vitro*. It has also been shown that DRG neurons in culture could be stimulated to axon growth after addition of VEGF_165_ (45). Our novel findings after dorsal root lesions of VEGF, NP1, and NP2 upregulation and the synchronous downregulation of SEMA3A are, as far as we know, the first possible indications of a VEGF-semaphorin interplay *in vivo*. If our findings of both NP1 and NP2 in combination with VEGF and in association with downregulation of SEMA3A and SEMA3F could reflect a VEGF-NP mediated regenerative machinery cannot be answered within the present study but is an interesting hypothesis.

In this study, we do also present findings on post-traumatic human DRG tissue. The findings might state that the anatomical distribution of SEMA3A, SEMA4F, and VEGF has been detected to have similar patterns in rat and man, and that the mRNA labeling intensity, can be compared to the levels documented in rat sections. One conclusion could be that these systems seem to react in similar ways in both rat and man.

The oligodendrocyte-derived extracellular matrix glycoprotein J1-160/180 (tenascin/J1 or janusin) is a recognition molecule expressed exclusively in the CNS. J1-160/180 has been shown to act as an attractant on astrocytes and repellent toward neurons and growth cones (51). The structural architecture predicted from the amino acid sequence is very similar to that of TN-R (52) and J1 should therefore probably be considered a TN-R isoform (53). Expression of J1 protein in the spinal cord is developmentally regulated, with a peak expression in 2- or 3-week-old animals. We have described the downregulation of mRNA for TN-R and J1 in spinal motoneurons after ventral funiculus lesion (14) and elevated J1 expression in the lesion area, after a cut in the ventral funiculus of the spinal cord (54). In the present study, we report that there were almost no changes in the labeling of mRNA for J1 after DRT compared to SNT and SNC, that both showed significant upregulation during the examined period making it complicated to conclude how J1 can be involved in the different regenerative responses described in these models.

In summary, we do in this study show regulatory patterns of the SEMA/NP-family and VEGF after injuries to the dorsal roots indicating an involvement in regenerative efforts of DRG neurites rather than inhibitory, which is puzzling regarding the supposedly unsuccessful regeneration of injured dorsal root sensory neurons. In addition, recent findings show that the regrowth of nerve roots into the spinal cord and the dorsal root entry zone can be supported under certain circumstances (4). This in summary might indicate that the findings in this study support that injured dorsal roots do have a regenerative capacity and that the regulatory patterns shown in this study is in fact part of the injured dorsal root ganglion cells effort to regenerate.

## Author Contributions

TL together with MR and MS conducted the main part of the practical laboratory work, surgery, and analysis of the results. HH and WW performed part of the surgery and analysis of sciatic injuries. TC contributed with the clinical material and analysis of the results. SC contributed to analysis of the study. MS supervised the work together with MR.

## Conflict of Interest Statement

The authors declare that the research was conducted in the absence of any commercial or financial relationships that could be construed as a potential conflict of interest. The reviewer FC declared a shared affiliation, though no other collaboration, with one of the authors MKS to the handling Editor, who ensured that the process nevertheless met the standards of a fair and objective review.
